# Supplementary material for: Early, sex-dependent and progressive proteomic imbalance in the amygdala during Alzheimer´s disease continuum
Source: Biol Sex Differ. 2026 May 29;17:139. doi: 10.1186/s13293-026-00930-9 (PMC13411746; doi:10.1186/s13293-026-00930-9)

# Supplemental Figure 1

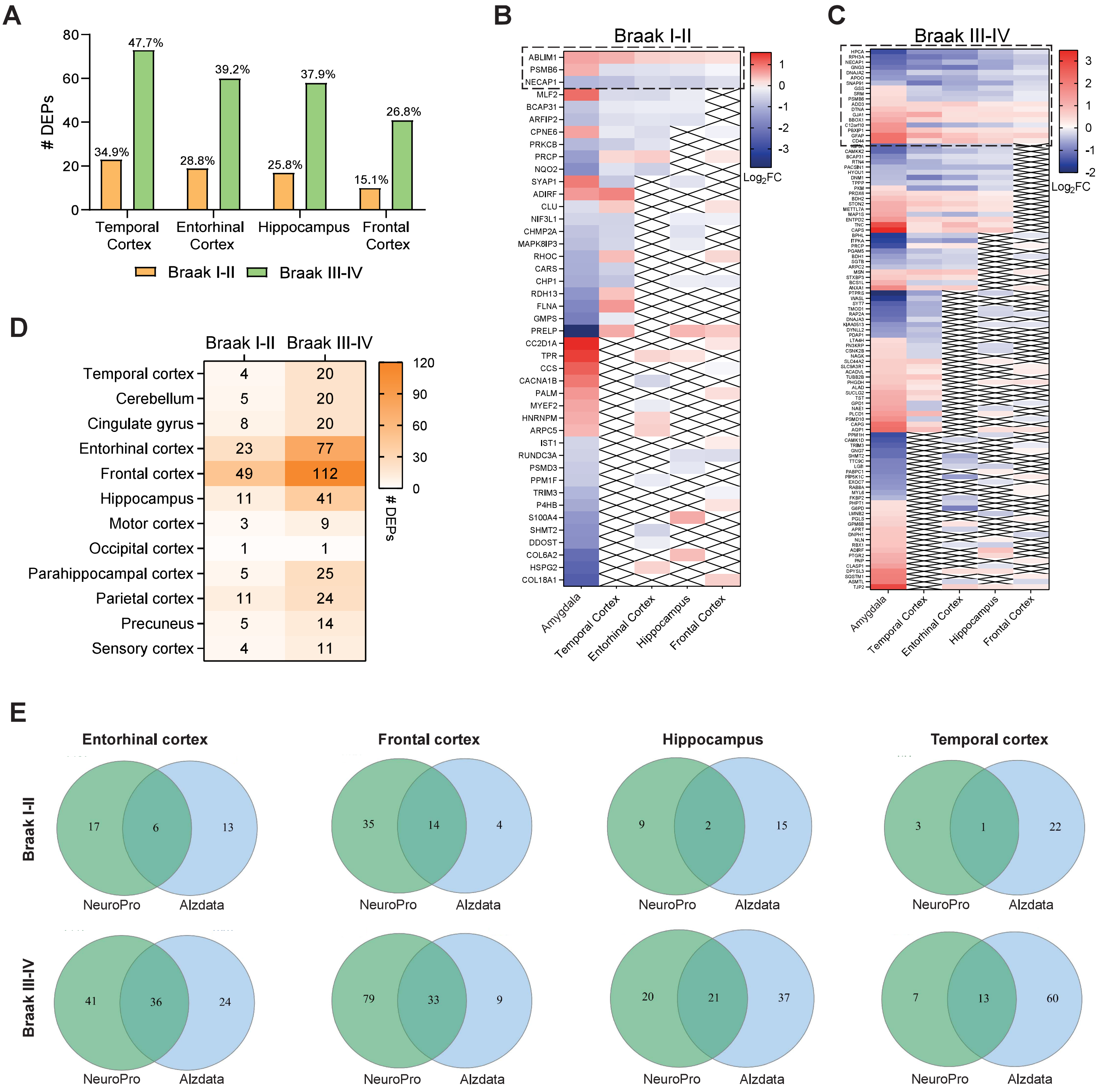

# Supplemental Figure 2

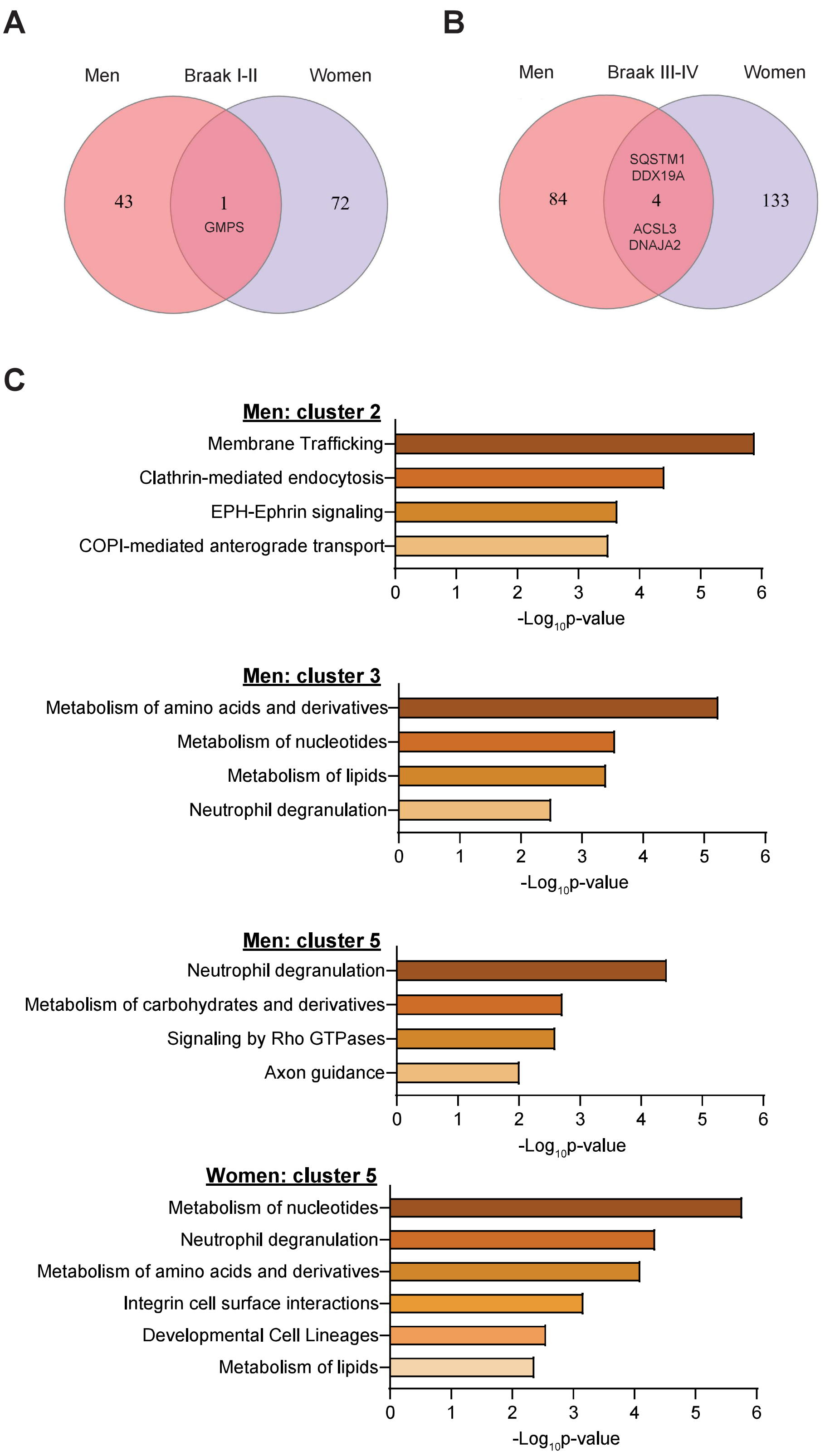

Supplement: Supplementary file 2 — Additional file 2. [file 13293_2026_930_MOESM2_ESM.pdf]
