# Supplementary material for: Early, sex-dependent and progressive proteomic imbalance in the amygdala during Alzheimer´s disease continuum
Source: Biol Sex Differ. 2026 May 29;17:139. doi: 10.1186/s13293-026-00930-9 (PMC13411746; doi:10.1186/s13293-026-00930-9)
Supplement: Supplementary file 3 — Additional file 3. [file 13293_2026_930_MOESM3_ESM.docx]

***Supplemental figure 1.* *Part of amygdaloid DEPs showed gene/protein expression correlation with AD in other brain regions****. (****A****) Percentage of proteins deregulated in Braak I-II and III-IV amygdaloid proteome that were also imbalanced at transcriptional level in other AD-affected regions published in Alzdata database [22,23]. Heatmap representing Log_2_Fold change of the proteins/genes differentially expressed in (****B****) Braak I-II or (****C****) Braak III-IV amygdala samples and other brain regions studied. (****D****) Number of common DEPs between Braak I-II or Braak III-IV amygdala proteome and 13 different brain regions from 38 published AD proteomic studies published in Neuropro database [24]. Dashed lines indicate common protein-coding genes between all brain areas studied. (****E****) Venn diagrams showing the overlap between* *the amygdala common DEPs deregulated in other brain regions (Neuropro) that were also altered at transcriptional level (Alzdata database). Figure created in* [*InteractiVenn.net*](https://www.interactivenn.net/) *[59].*

***Supplemental figure 2. Low overlap of DEPs between women and men.*** *Venn diagrams showing the overlap between* *the amygdala common DEPs deregulated in (****A****) Braak I-II and (****B****) Braak III-IV in men and women. Figure created in* [*InteractiVenn.net*](https://www.interactivenn.net/) *[59]. Reactome-based pathways significantly altered in derived from the deregulated proteins of the clustering analysis.*

***Supplemental Table 1.*** *Description of the amygdaloid samples included in this study.*

***Supplemental Table 2.*** *Quantified proteins detected in amygdaloid samples.*

***Supplemental Table 3.*** *Differential expressed proteins in amygdaloid Braak I-II stage compared to control group.*

***Supplemental Table 4.*** *Differential expressed proteins in amygdaloid Braak III-IV stage compared to control group.*

***Supplemental Table 5.*** *Common differentially expressed gene/proteins (p≤0.05) between Braak I-II or Braak III-IV amygdaloid datasets and other AD-affected brain regions from AlzData database [22,23] with their Log_2_Fold Change values.*

***Supplemental Table 6.*** *Common differentially expressed gene/proteins (p≤0.05) between Braak I-II or Braak III-IV amygdaloid datasets and other AD-affected brain regions from Neuropro database [24]. Positive and negative values indicate protein up- or down-regulation, respectively.*

***Supplemental Table 7.*** *Common differentially expressed gene/proteins (p≤0.05) between Braak I-II or Braak III-IV amygdaloid datasets and other AD-affected brain regions from AlzData and Neuropro databases [22–24].*

***Supplemental Table 8.*** *Differential expressed proteins in amygdaloid men Braak I-II stage compared to control group.*

***Supplemental Table 9.*** *Differential expressed proteins in amygdaloid men Braak III-IV stage compared to control group.*

***Supplemental Table 10.*** *Differential expressed proteins in amygdaloid women Braak I-II stage compared to control group.*

***Supplemental Table 11.*** *Differential expressed proteins in amygdaloid women Braak III-IV stage compared to control group.*

***Supplemental Table 12****. Clustering analysis separated by sex comparing controls, Braak I-II, and Braak III-IV subjects. Statistical analyses were performed by one-way ANOVA test.*

***Supplemental Table 13****. Commonly deregulated proteins (p<0.05) between Braak I-II or Braak III-IV amygdaloid datasets separated by sex and other AD-affected brain regions from Agora database. Values indicate Log2 Fold Change*

***Supplemental Table 14****. Top genes whose silencing reverse the proteomic signature of amygdala DEP datasets from each Braak stage. Connectivity scores near to -100 express highly potential activity to reverse the proteomic signature. Results were obtained from connectivity map database [20].*

***Supplemental Table 15****. Top genes whose silencing reverse the proteomic signature of amygdala DEP datasets from each Braak stage considering sex. Connectivity scores near to -100 express highly potential activity to reverse the proteomic signature. Results were obtained from connectivity map database [20].*
